# Effect of mindfulness on physical activity in primary healthcare patients: a randomised controlled trial pilot study

**DOI:** 10.1186/s40814-021-00810-6

**Published:** 2021-03-17

**Authors:** Peter Nymberg, Susanna Calling, Emelie Stenman, Karolina Palmér, Eva Ekvall Hansson, Kristina Sundquist, Jan Sundquist, Bengt Zöller

**Affiliations:** 1 Center for Primary Health Care Research, Region Skåne, Lund, Sweden; 2 Department of Clinical Sciences, Malmö Lund University, Lund, Sweden; 3 Department of Health Sciences/Physiotherapy, Lund University, Lund, Sweden

## Abstract

**Abstract:**

Increased physical activity can have health benefits among inactive individuals. In Sweden, the healthcare system uses physical activity on prescription (PAP) to motivate patients to increase their physical activity level. Mindfulness may further heighten the internal motivation to engage in physical activity. However, previous research has not demonstrated clear evidence of such an association.

**Aim:**

Examine the feasibility of the study design as a preparation for a full-scale study, and examine the differences, between three interventions, in change over time in physical activity levels and in related variables.

**Method:**

Comparison between three different interventions in an ordinary primary health care setting: PAP, mindfulness, and a combination of PAP and mindfulness. Physical activity was measured with self-report and ACTi Graph GT1X activity monitor. Statistical analysis was performed with a mixed-effect model to account for repeated observations and estimate differences both within groups and between groups at 3- and 6-months follow-up.

**Results:**

Between September 2016 and December 2018, a total of 88 participants were randomised into three groups. The total dropout rate was 20.4%, the attendance rate to the mindfulness courses (52% > 6 times) and the web-based mindfulness training (8% > 800 min) was low according to the stated feasibility criteria. Eleven participants were excluded from analysis due to low activity monitor wear time. Neither the activity monitor data nor self-reported physical activity showed any significant differences between the groups.

**Conclusion:**

The study design needs adjustment for the mindfulness intervention design before a fully scaled study can be conducted. A combination of PAP and mindfulness may increase physical activity and self-rated health more than PAP or mindfulness alone.

**Trial registration:**

ClinicalTrials.gov, registration number NCT02869854. Regional Ethical Review Board in Lund registration number 2016/404.

**Supplementary Information:**

The online version contains supplementary material available at 10.1186/s40814-021-00810-6.

## Key messages regarding feasibility

- What uncertainties existed regarding the feasibility?
  An association between being mindful and physical activity level has been suggested. However, it is still unknown whether it is mindfulness that leads to increased physical activity or vice versa. Physical activity on prescription (PAP) is an established method for promoting physical activity and we wanted to examine the feasibility of comparing sedentary primary care patients that were randomized into PAP, mindfulness training or a combination of both. Feasibility was estimated by measuring adherence, recruitment rate and number of dropouts. The results will be used to design a larger scale randomized controlled study with a proper power calculation.
- What are the key findings?
  Of 136 eligible patients, a total of 88 volunteered to participate thus resulting in a recruitment rate of *64.7%.* Only 52% of the patients, which were randomized to only mindfulness or the combination group, attended the mindfulness course, and only *8%* did 800 min or more of the associated web-based training. Besides the low dropout rate of 20.4%, there was loss to follow-up regarding accelerometer data. There was a tendency towards an increased effect of PAP on physical activity level when mindfulness training was added.
- What are the implications of the feasibility findings for design of the main study?
  The mindfulness intervention needs to be remoulded to improve compliance in a larger-scale study. The type of accelerometers used (hip/wrist worn) also need to be considered to minimize the loss of objective physical activity data to follow-up.

## Introduction

A lifestyle with an adequate amount of physical activity can decrease the risk of both somatic and mental illness [1–4]. Although people in northern Europe are generally physically active [5], they report more sedentary time than their southern European counterparts [6]. A study regarding sedentary behaviour, among 50- to 64-year-old Swedish adults, showed that only 7.1% of the 948 participants fulfilled the World Health Organization’s (WHO) recommendation for physical activity [7]. Making individuals change their physical activity behaviour is an ongoing challenge. A systematic review estimated that 12 sedentary adults need to be treated with a physical activity promotion intervention in order to make one of them achieve the recommended physical activity level at one year follow-up [8]. In Sweden, the health care services recommend the use of physical activity on prescription (PAP) as a complementary treatment to motivate patients to increase their physical activity level; the treatment addresses both primary and secondary prevention of illness. The written prescription in the Swedish PAP model can be a proposal for an activity or an extensive solution with a supportive structure depending on the patient’s needs and level of motivation. The Swedish PAP model has been associated with up to 60% increased activity levels, but the effect has not shown sustainability over time [9–11]. In some of the studies complemented with pedometers, the most common way to report the effect of PAP has been by self-reported measures [11]. To the authors’ knowledge, only one study has used activity monitors to measure the effect of the Swedish PAP model [12] and it failed to show significantly increased levels of moderate to vigorous physical activity (MVPA).

Research has demonstrated that satisfaction can play a crucial role in changing a behaviour such as physical activity [13], smoking cessation [14] or weight loss [15]. It has been suggested that satisfaction can be increased both by the awareness in a specific positive situation and by the reduction of negative thoughts, e.g. about physical activity [16]; something which may be facilitated by practising mindfulness [17]. All people have a varying intrinsic, albeit modifiable, trait to be aware of the present moment—dispositional mindfulness [18]. Mindfulness can be exerted as sitting meditation but also as an approach to everyday life [19]. The practice of mindfulness might give the individual an orientation toward one’s experiences in the present moment [20]. Being mindful in a specific situation and, in addition, satisfaction are suggested to be consecutive mediators for the path between possessing a dispositional tendency to be mindful and physical activity [17]. This might explain why self-reported mindfulness seems to mediate the relationship between intrinsic motivation and the physical activity level [21]. In addition, mindfulness has been shown to increase pain tolerance [22, 23]. In other words, practising mindfulness can make it easier to experience satisfaction and mitigate discomfort connected with physical activity and, in this way, support the change from a physically inactive to a physically active behaviour. Recent research has shown a lower decrease—if mindfulness was being practised—in physical activity due to seasonal decline compared with a control group [24]. A review, which was conducted to investigate the role of mindfulness in physical behaviour changes, revealed that mindfulness interventions influenced the physical activity outcomes in a positive direction in a majority of the 40 included studies [25]. Mindfulness-based interventions were suggested to be successful if they targeted psychological factors related to increased physical activity, such as self-efficacy and acceptance. Even if mindfulness correlates with factors that can influence the increase of physical activity, it has been shown that regular exercise can lead to an increased dispositional mindfulness [26]. Thus, mindfulness may have an important role to play concerning motivation by reinforcing satisfaction with physical activity [25].

## Aim

To examine the feasibility of the study design as a preparation for a full-scale study.

The intervention outcome was differences of change in physical activity level over time between three groups: PAP, mindfulness and a combination containing both PAP and mindfulness, in a population with insufficient self-reported levels of physical activity.

## Methods/design

### Participants

This was a pilot-study preparing for a larger-scale randomised trial. For detailed information about the study, we refer to the published study-protocol [27]. Men and women, which could speak fluent Swedish and were aged between 40 and 65 years, visiting their primary healthcare clinic for any reason were asked to rate their physical activity level. Those with a self-rated physical activity level below the WHO recommendations were asked to participate in the study. We excluded from the study (within 6 weeks before study entry) those with dementia, severe mental disorder, unstable untreated angina pectoris or myocardial infarction. The criteria of physical activity were defined according to the WHO guidelines, in which the lower limits for sufficient activity are set to 150 min per week of moderate intensity or 75 min per week of high intensity exercise.

**Table 1:** Patient characteristics for all participants at baseline, 3 months and 6 months

Randomisation group	PAP	Mindfulness	Combination
**Baseline** *n* (%)			
Women	22 (76)	22 (73)	20 (69)
Men	7 (24)	8 (27)	9 (31)
**3 months**			
Women	18 (72)	19 (79)	17 (65)
Men	4 (18)	5 (21)	9 (35)
**6 months**			
Women	17 (77)	19 (79)	15 (63)
Men	5 (23)	5 (21)	9 (37)
**Age** median (range)			
Baseline	54 (42–65)	53 (41–65)	54 (43–64)
**Percent of time in sedentary time**^a^ mean (SD, range)			
Baseline	66.2 (6.7, 78.2–50.2)	65.5 (9.2, 80.3–43.7)	66.7 (8.8, 80.0–47.2)
3 months follow-up	66.7 (6.7, 78.3–56.3)	65.5 (7.5, 78.9–51.3)	67.5 (8.1, 81.2–51.3)
6 months follow-up	65.0 (6.8, 73.6–50.0)	65.9 (9.4,81.0–36.2)	64.0 (9.1,78.4–45.6)
**Percent of time in LIPA**^**a**^ mean (SD, range)			
Baseline	30.9 (6.2, 45.5–20.9)	31.7 (8.2, 50.0–19.0)	30.3 (8.0, 49.2–18.8)
3 months follow-up	31.0 (6.0, 42.6–21.5)	31.8 (6.9, 47.8–19.9)	28.6 (7.1,44.5–16.3)
6 months follow-up	32.0 (5.8, 43.8–23.1)	31.6 (9.0, 58.4–17.0)	32.8 (8.9, 50.7–18.6)
**Percent of time in MVPA**^a^median (SD, range)			
Baseline	2.6 (0.18, 6.3–0.5)	2.3 (0.02, 9.1–0.1)	2.4 (0.02, 9.0–0.2)
3 months follow-up	2.4 (0.14, 5.0–0.2)	1.9. (0.02, 12.7–0.3)	3.4 (0.03, 11.1–0.1)
6 months follow-up	2.6 (0.02, 6.2–0.4)	1.9 (0.02, 9.0–0.2)	2.7 (0.02, 9.2–0.3)
**Weight kg** median (range)			
Baseline	92.0 (67–121)	86.8 (57–132)	81.5 (62–146)
3 months follow-up	*87.9 (67–111)*	81.2 (56–135)	83 (62–143)
6 months follow-up	*91.3 (59–110)*	83.0 (60–139)	80.2 (60–145)
**BMI kg/m**^**2**^ median (range)			
Baseline	31.4 (21–43)	29.9 (22–44)	28.5 (21–40)
3 months follow-up	30.0 (21–42)	28.6 (22–43)	28.2 (21–39)
6 months follow-up	29.4 (21–42)	28.6 (23–44)	27.9 (23–39)
**Total cholesterol mmol/L** mean (SD, range)			
Baseline	5.20 (1.05, 7.7–2.9)	5.63 (0.87, 7.3–4.1)	5.41 (1.04, 7.4–3.4 )
3 months follow-up	4.99 (1.02, 6.9–3.3)	5.35 (0.96, 7.1–3.8)	5.12 (1.00, 7–3.1)
6 months follow-up	5.08 (1.23, 7.1–3)	5.72 (1.09, 7.7–3.6)	5.08 (1.19, 7.6–2.9)
**Low-density cholesterol mmol/L** mean (SD, range)			
Baseline	3.43 (1.04, 5.9–1.3)	3.80 (0.92, 5.7–2)	3.66 (0.94, 5.5–2)
3 months follow-up	3.15 (0.97, 5.1–1.7)	3.51 (1.02, 5.8–2)	3.52 (1.01, 5.3–1.7)
6 months follow-up	3.26 (1.14, 5.3–1.5)	3.83 (1.04, 5.9–1.9)	3.57 (1.15, 6.3–1.6)
**High-density cholesterol** **mmol/L** median (SD, range)			
Baseline	1.5 (0.54, 3.1–0.9)	1.4 (0.52, 2.9–0.5)	1.5 (0.36, 2.4–1.0)
3 months follow-up	1.5 (0.56, 2.7–0.9)	1.5 (0.60, 2.8–0.4)	1.5 (0.32, 2.1–0.9)
6 months follow-up	1.5 (0.55, 2.7–0.9)	1.4 (0.64, 2.9–0.4)	1.4 (0.27, 2.3–0.9)
**Triglycerides mmol/L** median (SD, range)			
Baseline	1.2 (0.97, 5.1–0.4)	1.6 (1.14, 5.2–0.5)	1.4 (0.70, 3.2–0.7)
3 months follow-up	1.25 (0.68, 3–0.5)	1.4 (1.13,4.9–0.8)	1.5 (0.62, 3.2–0.7)
6 months follow-up	1.3 (0.87, 4.1–0.4)	1.65(1.18, 5.8–0.5)	1.4 (0.60,3.2–0.7)
**Systolic blood pressure** **mmHg** median (range)			
Baseline	130 (110–160)	120 (80–160)	130 (100–155)
3 months follow-up	127 (100–150)	122 (104–165)	120 (106–145)
6 months follow-up	126 (90–160)	129 (102–150)	131 (108–160)
**Diastolic blood pressure** **mmHg** median (range)			
Baseline	80 (64–100)	80 (60–100)	80 (60–90)
3 months follow-up	80 (60–90)	80 (68–99)	78(60–90)
6 months follow-up	80 (60–100)	80 (60–100)	80 (60–100)
**Leisure-time activity**^b^ (min = 0, max = 6) median (range)			
Baseline	1 (1–5)	2 (1–3)	2 (1–4)
3 months follow-up	3 (1–6)	2 (1–3)	3 (1–6)
6 months follow-up	3 (1–6)	2 (1–6)	3 (1–6)
**Daily activity**^b^(min = 0, max = 7) median (range)			
Baseline	3 (1–7)	3 (1–6)	4 (1–5)
3 months follow-up	5 (2–7)	3 (1–7)	5 (1–6)
6 months follow-up	5 (3–7)	4 (1–7)	5 (1–7)
ISI^b^ (min = 0, max = 28) median (range)			
Baseline	8 (0–24)	11 (0–27)	10 (0–21)
3 months follow-up	7 (0–20)	11 (0–27)	9 (0–27)
6 months follow-up	6 (0–18)	13 (0–25)	9 (0–18)
FFMQ^b^ (min = 29, max = 145) mean (SD)			
Baseline	105.5 (80–129)	105.6 (82–128)	100.6 (89–114)
3 months follow-up	107.6 (90–125)	103.5 (81–129)	103.8 (92–117)
6 months follow-up	106.8 (85–132)	102.5 (83–127)	102.8 (87–116)
SRH^b^ (min = 1 max = 5) median (range)			
Baseline	3 (1–4)	3 (2–5)	3 (2–5)
3 months follow-up	3 (1–4)	3 (2–5)	4 (2–5)
6 months follow-up	4 (1–4)	4 (2–5)	4 (2–5)

^a^Percentage of sedentary time and time in different intensity of physical activity per valid day and averaged over the number of valid days, activity monitor measured

^b^Self-reported measurements. Data are presented as mean values and standard deviation (SD) for normally distributed variables, and as median and range for variables with skewed distribution and variables based on nominal scales. *LIPA* light physical activity, *MVPA* moderate to vigorous physical activity, *ISI* insomnia severity index, *FFMQ* five facets of mindfulness questionnaire, *SRH* self-rated health

### Setting

The pilot study involved three primary health care centres, recruited on voluntary basis, in the county of Scania in southern Sweden. In total, there are approximately 164 primary health care centres in the county. Scania has approximately 1.2 million residents and about 400,000 of these are aged between 40 and 65 years old.

### Outcome measures

#### Feasibility criteria for a successful design

1. Recruitment rate more than 30% [28].
2. Dropout rate of less than 30%.
3. Compliance to the mindfulness course: ≥ 70% of those randomised to any of the groups containing mindfulness should participate in at least 75% of the mindfulness meetings [29].
4. Web-based practice for at least 20 min 5 days per week (800 min) by 70% of patients randomised to any group containing mindfulness [30].

#### Intervention outcome

All measurements and questionnaires were collected at baseline, after 3 months and after 6 months.

The primary intervention outcome was changed level of physical activity, self-reported and measured by ACTi Graph GT1X activity monitors. We used the same definitions and methods to handle the activity monitor data as in previously published research regarding physical activity [7, 12]. Activity monitor data were divided into sedentary, light physical activity (LIPA) and moderate to vigorous physical activity (MVPA). The participants were instructed to wear the activity monitor every day for a week before randomisation, at 3 months and at 6 months follow-up. Wear time was defined by subtracting non-wear time from 24 h. Non-wear time was defined as at least 60 consecutive minutes with no movement (0 counts per minute), with allowance for maximum 2 min of counts between 0 and 100 [7, 31]. We considered ≥ 600 min wear time per day for at least 4 days to be valid compliance [7, 12]. Due to a small sample size, we did not demand four consecutive days with valid wear time, and we did not differ between weekdays and weekends. Average was expressed as total counts divided by wear time in minutes per day (counts per minute) and averaged over worn days. Registrations below 100 counts per minute were determined as being sedentary [7, 12, 32]. 100–2019 counts per minute were considered as LIPA and > 2020 counts per minute as MVPA [7, 12]. The results are presented as percentage sedentary, LIPA or MVPA per valid day and averaged over the number of valid days [12] self-reported daily activity (e.g. gardening, slow walks, biking) was measured by an eight-step scale (0 = 0 min/week, 7 ≥ 300 min/week), and self-reported leisure time activity (e.g. running, football) was measured by a seven-step scale (0 = 0 min/week, 6 ≥ 200 min/week).

#### Secondary intervention outcomes

1. Change in self-rated health (SRH) between baseline and follow-up, measured with a five-step scale (1–5): very poor, poor, fair, good or very good.
2. Change in blood pressure, weight or serum lipids between baseline and follow-up.
3. Change in insomnia problems as measured with insomnia severity index (ISI) [33] between baseline and follow-up.
4. Change in mindfulness measured with five facets of mindfulness questionnaire (FFMQ) [34] between baseline and follow-up.

### Interventions

Participants in the PAP group were prescribed Swedish PAP [9, 35], which is the recommended treatment for physically inactive patients and adjusted to each patient’s individual preferences.The participants in the mindfulness group received a two-hour long mindfulness group session once a week for 8 weeks and were instructed to practise mindfulness for 20 min every day. The mindfulness course [29] was based on both Mindfulness-Based Stress Reduction (MBSR) and Mindfulness-Based Cognitive Therapy (MBCT) and included meditative exercises. The patients received instructions concerning the daily mindfulness practice with meditative exercises via a web-based program [29]. The instructions included breathing technique and body scan.The combination group comprised both PAP and mindfulness, meaning an individually adjusted PAP combined with an addition of the same mindfulness course as in the mindfulness group.

### Statistics

#### Power calculation

Sample size of a full-scale intervention study with a follow-up time at 12 months was calculated on a 1:1 relationship between two groups (PAP and combination), and estimated to *n* = 375 in each group, based on a power analysis with 5% significant-level and a power of 80%. Drop out was expected to be 30%. The calculation was based on other studies with self-reported compliance to PAP as an outcome measure, where 50% of the participants followed the recommendation on physical activity from the PAP. We estimated an increase from 50% to 62.5% of self-reported adherence to PAP [36]. The pilot-study sample size is based on the assumed patient flow and due to a limited project budget [37]. In this pilot study, we aimed to include approximately 30 participants in each arm, which is in concordance to a general flat rule, using a minimum of 30 participants to be able for estimating a parameter [38].

#### Randomisation

The randomisation to the three intervention groups (PAP, combination, mindfulness) was stratified by the patients’ age and sex, with a total of three age groups: 40–49, 50–59 and 60–65 years. The randomisation was done by a minimisation method with a random element, as minimisation variables in the randomisation we used age and sex to get the groups as equal as possible [39]. The randomisation was done in the statistical programme STATA version 15 (StataCorp, College Station, TX).

#### Statistical methods

The intervention effect on changes in outcome measures was examined by analysing average group differences (PAP, combination, mindfulness) in baseline score and change in each outcome between baseline and 3 and 6 months follow-up using a linear mixed-effects model. Each model included the time variable and group as indicator variables, and an interaction between time and group to estimate treatment differences in change over time, adjusted for baseline measures and taking the correlation between repeated measurements into account. We did not adjust for the minimisation variables in the analysis. Statistical analyses were done using STATA version 15 (StataCorp, College Station, TX).

## Results

### Recruitment

**Fig. 1:**
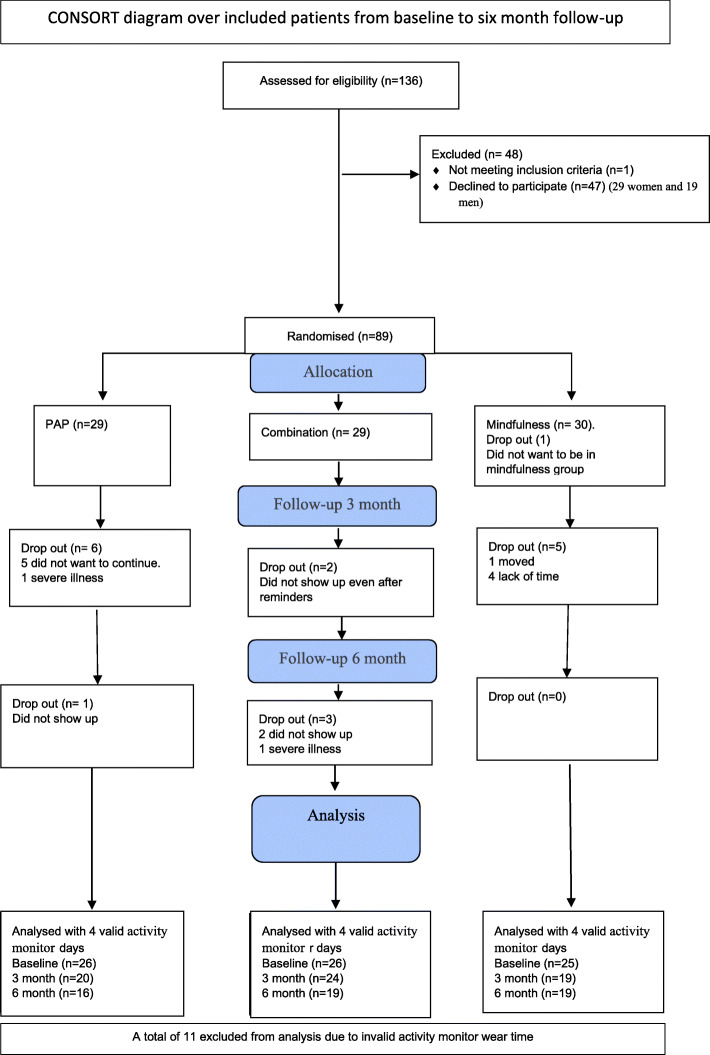
CONSORT diagram over included patients from baseline to 6-month follow-up

For the period 1st of September 2016 until the 31st of December 2018, a total of 136 eligible patients were asked to participate in the study and 88 were included. The median age among the participants in the PAP-group was 54 years; in the combination-group, it was 54 years and in the mindfulness-group it was 53 years (Table 1). Among those who declined participation, the average age was 56 years (29 women and 19 men) (Fig. 1).

### Fulfilment of feasibility criteria

We monitored several feasibility criteria to evaluate the suitability of the study design [27]. If all the feasibility criteria were fulfilled, the main study was considered possible to conduct without further changes in the protocol. If the criteria were not fulfilled, the protocol was considered to need adjustment, and if the criteria were fulfilled to less than 70% it was considered not possible to carry on with a full-scale study in the current form.

A recruitment rate of 30% was considered to be successful; 88 (64.7%) of all the 136 patients eligible for the study, who were asked to participate, accepted.

A dropout rate of less than 30% was considered successful: during the study, there were a total of 20.4% (5 men, 13 women) dropouts with an average age of 52.

A successful attendance rate to the mindfulness course was set to ≥ 70%, of those randomised to any of the groups containing mindfulness should participate in at least 75% of the mindfulness meetings. There were 52% (*n* = 15 in the mindfulness group, *n* = 16 in the combination) who attended six times or more to the meetings.

Seventy percent of patients randomised to any group containing mindfulness should practise mindfulness for at least 20 min with the web-based application at least 5 days a week (a total of 800 min or more). The mean time spent in web-based training, during the study, was 184.69 min with a standard deviation of 330.93 min (minimum 0 and max 1300 min). Only 8% (seven persons) did 800 min or more (*n* = 4 in the mindfulness group, *n* = 3 in the combination).

#### Secondary outcome

The patients were randomised into the three groups, PAP (*n* = 29), combination (*n* = 29), or mindfulness (*n* = 30). In the PAP-group, there were 24.1% (2 men, 5 women) dropouts with an average age of 53 years. In the mindfulness group, there were 20% (3 men, 3 women) dropouts with an average age of 50 years. The combination group had 17.2% (5 women) of dropouts with an average age of 53 years (Fig. 1). Two dropouts were due to illness, five persons did not show up at follow-ups even after two reminders. Six individuals did not want to continue without giving any reason, four people cited lack of time and one person moved and could not continue participating in the study (Fig. 1). After exclusion of those with fewer than four valid activity monitor wear days, *n* = 26 in the PAP-, *n* = 26 in the combination- and *n* = 25 in the mindfulness group remained. The wear time with activity monitors differed between 0 and 12 days. There were no significant baseline differences between the dropouts and the remaining participants (see Additional file 1). There were over 80% of the participants at each time-point who wore the accelerometer for 4 days or more (see Additional file 2)

In the sensitivity analyses, we analysed the data in several different ways, both with one valid activity monitor day (see Additional file 3), and four valid activity monitor days (Table 2), with similar results.

**Table 2:** Intercept (adjusted baseline value) and changes from baseline to 3 and 6 months in the three groups using mixed-effect models. Individuals with at least 4 valid days (600 min activity monitor wear time per day)

Outcome	Adjusted baseline value	Change from baseline to 3 months	Change from baseline to 6 months	Overall mean difference between groups over time^d^ (95% CI)
Sedentary ^a^ (percentage)				− 0.75 (− 1.74 ; 0.22)
PAP	66.3	0.5	0.2	
Mindfulness	65.8	1.1	1.4	
Combination	66.4	− 0.2	− 2.8	
LIPA ^a^ (percentage)				0.56 (− 0.35 ; 1.47)
PAP	30.9	0.01	0.03	
Mindfulness	31.7	− 1.1	− 1.3	
Combination	30.5	− 0.9	2.4	
MVPA^a^ (percentage)				0.15 (− 0.19 ; 0.48)
PAP	2.9	− 0.5	− 0.2	
Mindfulness	2.5	− 0.1	− 0.1	
Combination	3.1	1.0	0.2	
Leisure time activity (1–6) ^b^				0.23 (− 0.03; 0.49)
PAP	1.67	1.11	1.18	
Mindfulness	1.88	− 0.05	0.59	
Combination	1.81	1.62	2.00	
Daily activity (1–7) ^b^				1.93 (− 0.22; 0.26)
PAP	3.40	1.21	1.09	
Mindfulness	3.63	0.39	0.77	
Combination	3.42	1.16	1.14	
Weight (kg)				0.18 (− 0.32; 0.68)
PAP	90.9	− 1.8	− 2.4	
Mindfulness	85.3	− 0. 13	− 0.6	
Combination	84.8	− 0.74	− 1.6	
SRH^c^ (1–5)				0.8 (− 0.06; 0.21)
PAP	3.2	0.2	0.2	
Mindfulness	3.3	0.3	0.3	
Combination	3.3	0.4	0.5	

^a^Percentage of mean time measured by activity monitor, *LIPA* light physical activity, *MVPA* moderate to vigorous physical activity

^b^Self-reported measurements: leisure time activity on a scale from 0 = 0 min per week, 6 ≥ 120 min/week Daily activity on a scale 0 = 0 min per week, 7 ≥ 300 min per week

^c^Self-rated health

^d^interaction between all three groups and timepoints

### Intervention outcomes

#### Differences in change between intervention groups

**Fig. 2:**
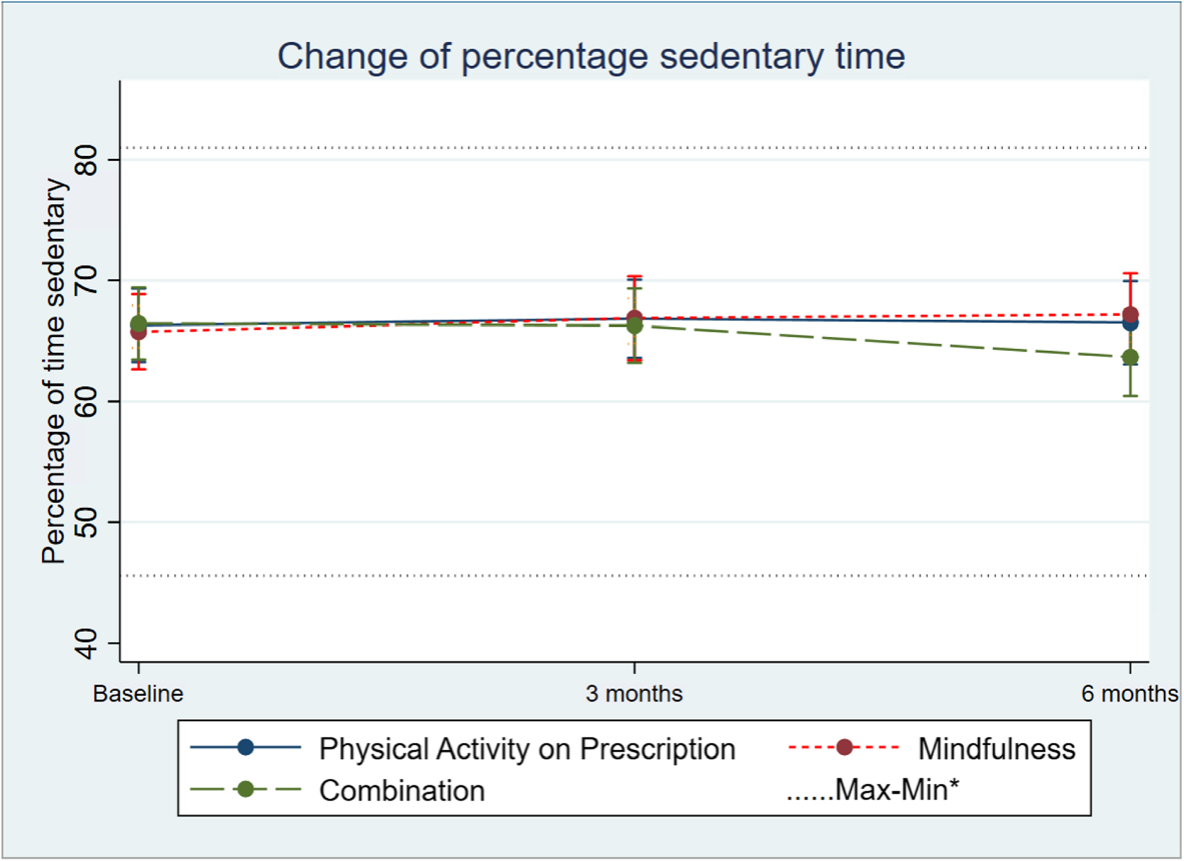
Change in percentage sedentary in 1 the three groups over time. Differences between and within the groups are estimated by a mixed-effect model. *Maximum and minimum value of all observations

**Fig. 3:**
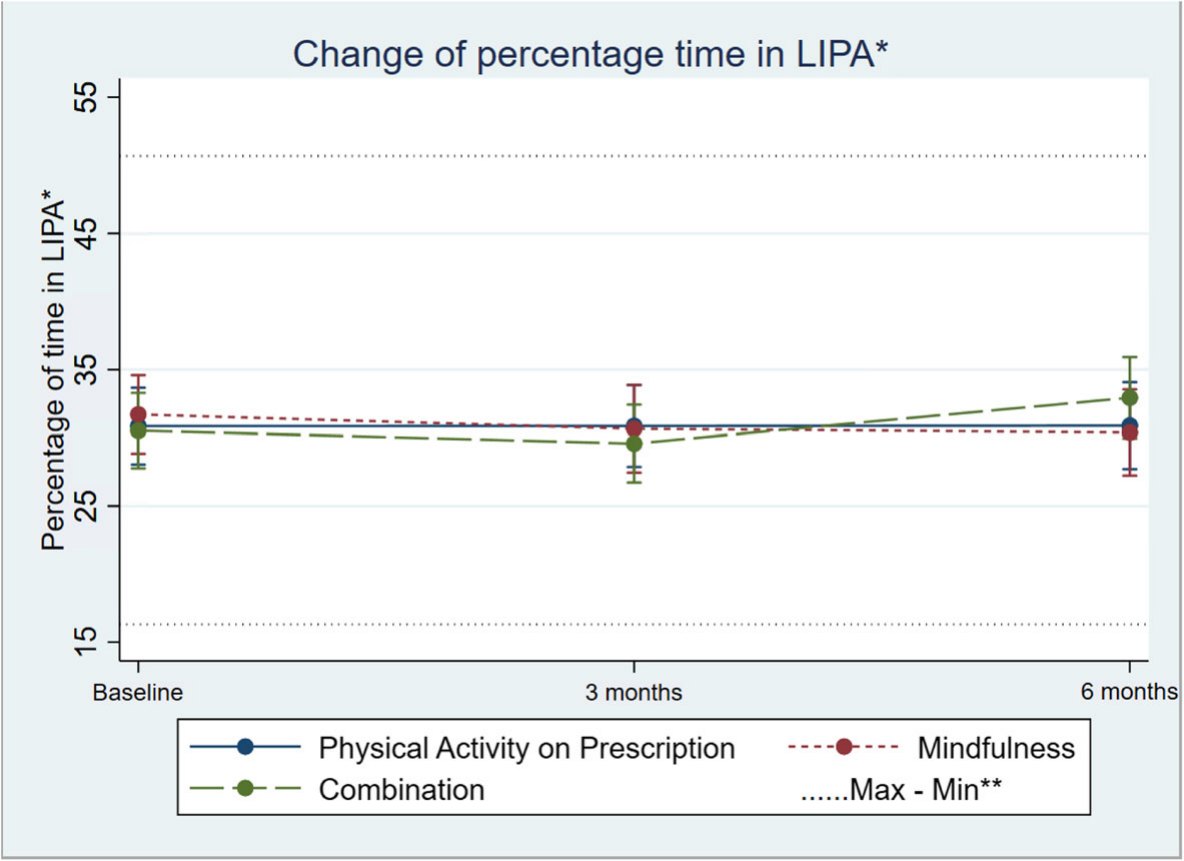
Change in percentage light physical activity (LIPA), in the three groups over time. Differences between and within the groups are estimated by a mixed-effect model. **Maximum and minimum value of all observations

**Fig. 4:**
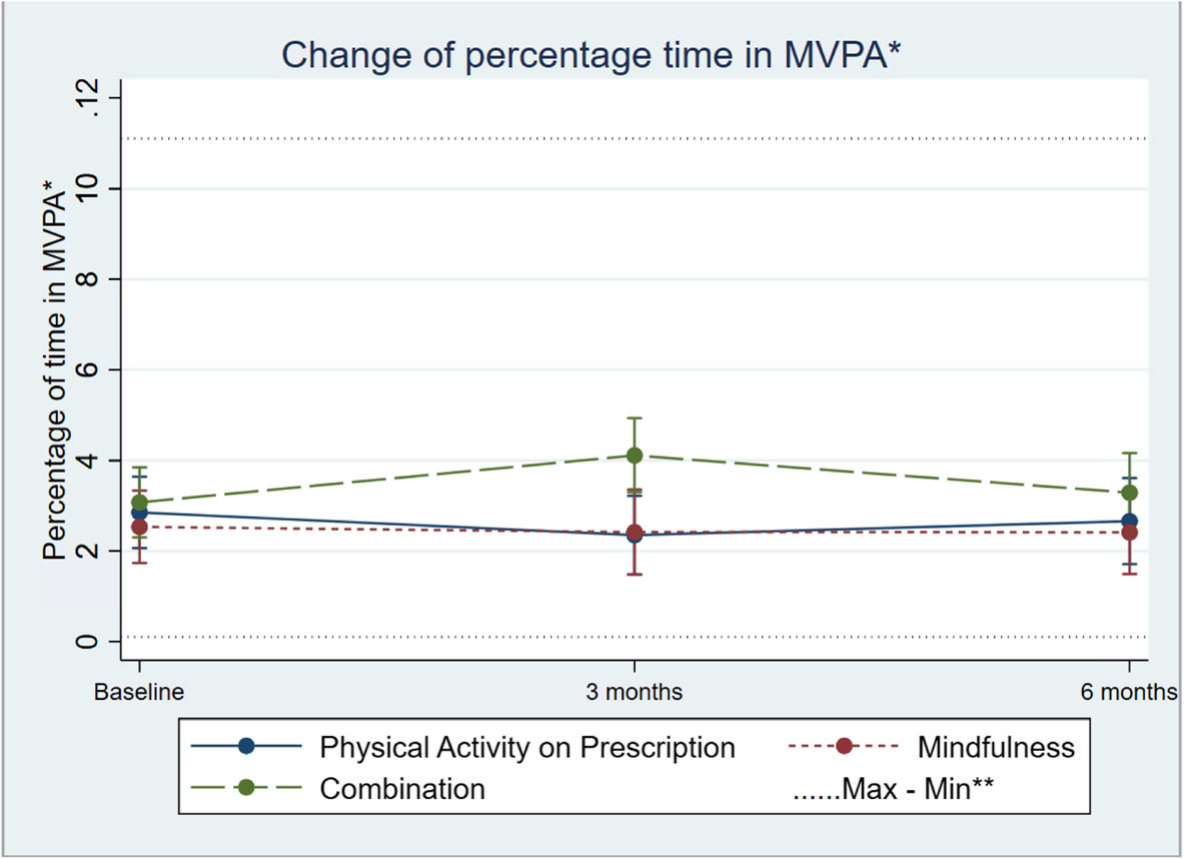
Change in percentage moderate to vigorous physical activity (MVPA), in the three groups over time. Differences between and within the groups are estimated by a mixed-effect model. *Moderate to vigorus physical activity. **Maximum and minimum value of all observations

Regarding group differences in alteration over time, percentage sedentary time showed only small and non-significant indications of differences between the three groups (95% CI − 1.74 ; 0.22; Table 2; Fig. 2). The same signals of suggested alteration were seen in differences regarding change in mean percentage of time in LIPA (95% CI − 0.35; 1.47; Table 2; Fig. 3) between the three groups. There was only a minor alteration in mean percentage of time in MVPA, and no differences in change between the three groups (95% CI − 0.19; 0.48; Table 2; Fig. 4).

**Fig. 5:**
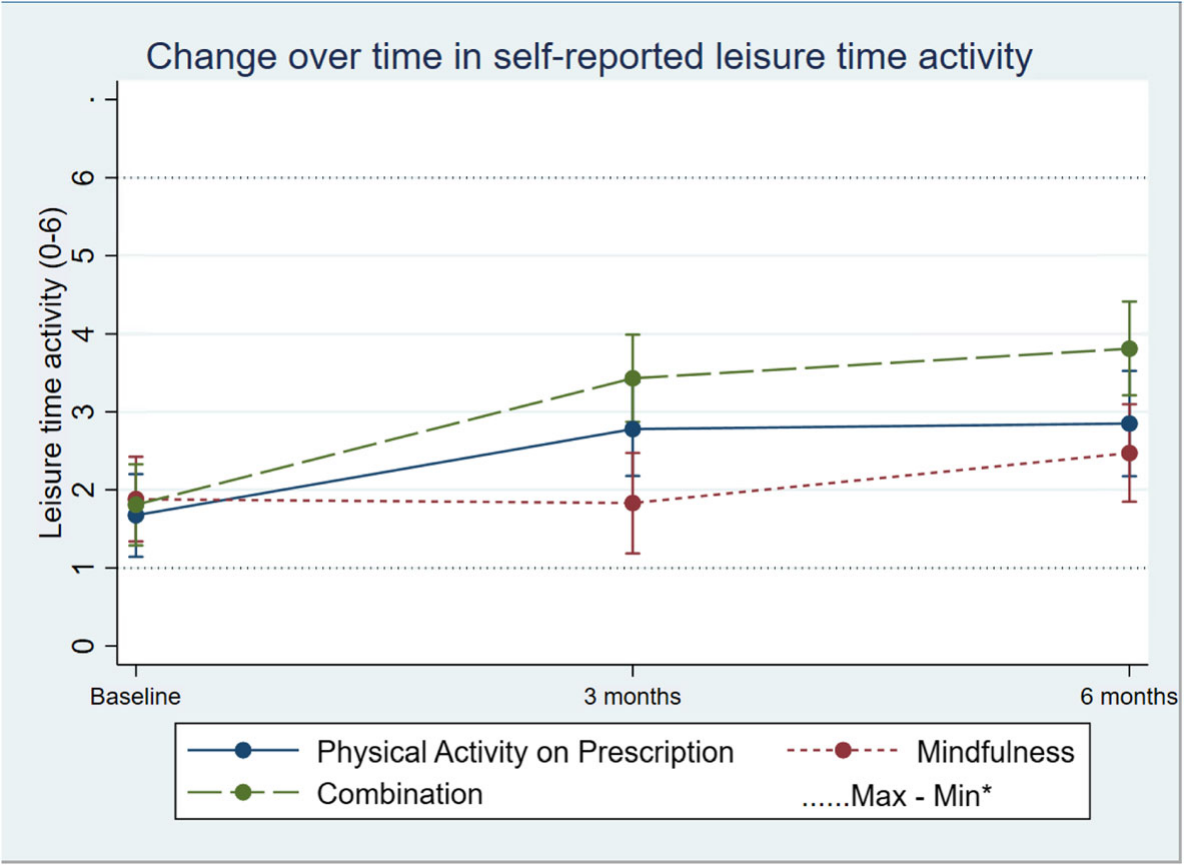
Change in units of self-reported leisuretime activity in the three groups over time. Differences between and within the groups are estimated by a mixed-effect model. *Maximum and minimum value of all observations

**Fig. 6:**
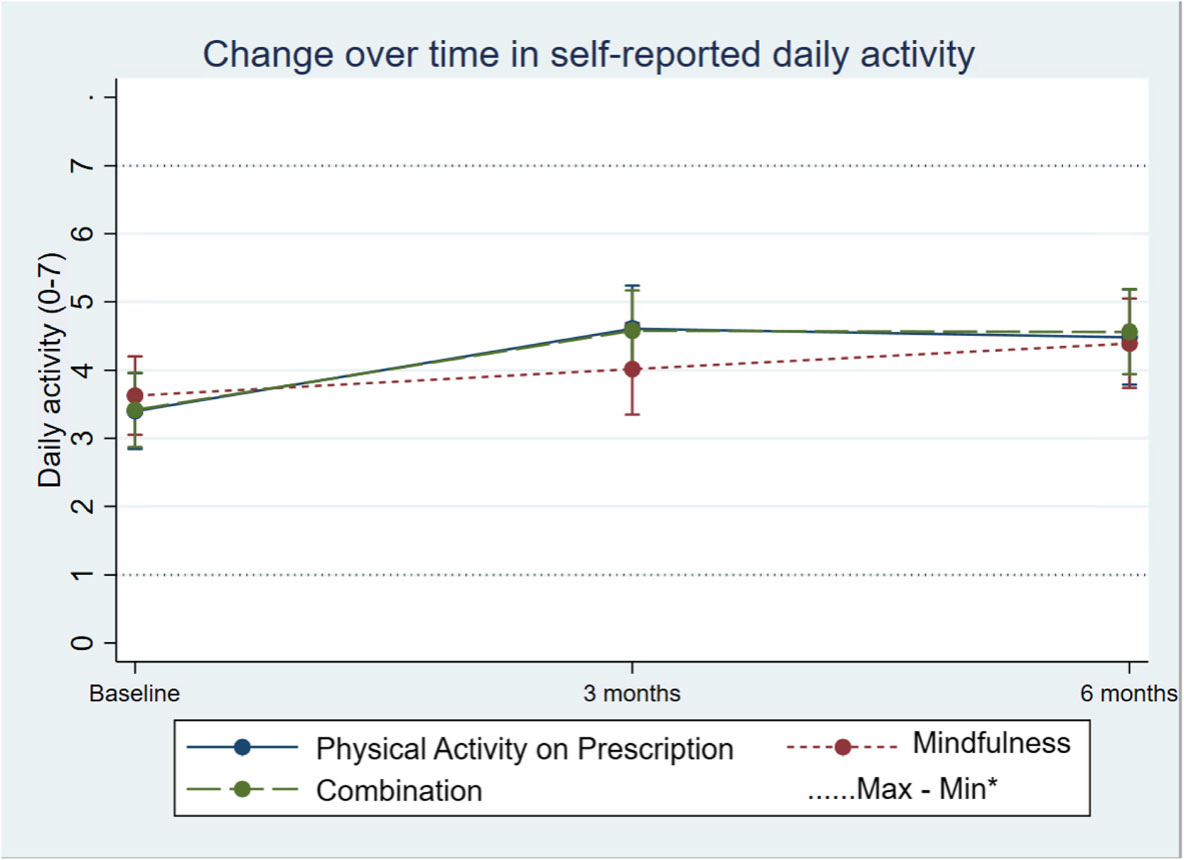
Change in units of self reported daily activity in the three groups over time. Differences between and within the groups are estimated by a mixed-effect model. *Maximum and minimum value of all observations

Self-reported leisure time activity increased in all groups but did not show any overall difference in change (95% CI − 0.03; 0.49) between the groups (Table 2; Fig. 5). The same pattern was seen in self-reported daily activity, with no difference in change between groups over time (95% CI − 0.22; 0.26; Table 2; Fig. 6).

#### Secondary intervention outcomes

**Fig. 7:**
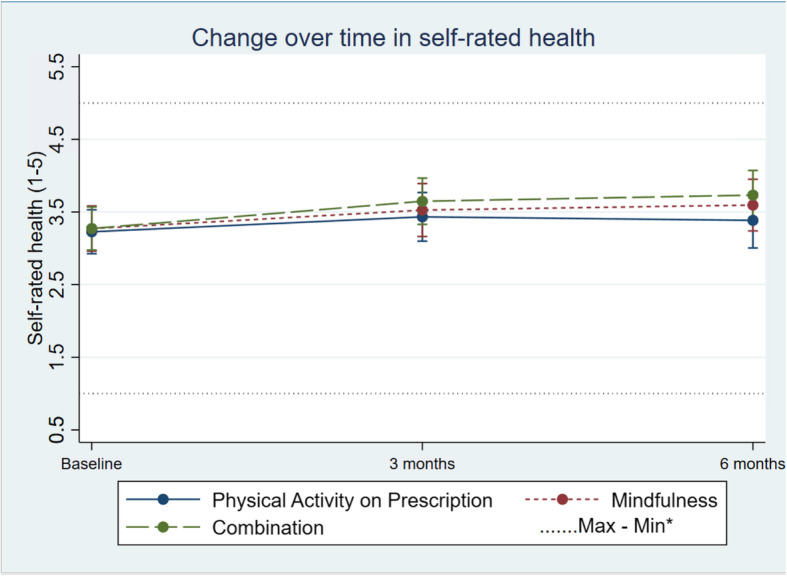
Change in units of self-rated health in the three groups over time. Differences between and within the groups are estimated by a mixed-effect model. *Maximum and minimum value of all observations

The analysis did not show any indications of large differences in change between groups regarding SRH (Table 2; Fig. 7), ISI (see Additional file 4) or FFMQ (see Additional file 4). The indicated alteration of blood pressure (see Additional file 4), weight (Table 2; figure see Additional file 5), BMI (see Additional file 4 for table, see Additional file 6 for figure) and blood lipids (see Additional file 4) did not suggest any statistically significant differences in change over time between the three groups

.

## Discussion

### Main findings of the study

#### Feasibility

Since only two of the four feasibility criteria were fulfilled, major changes in the mindfulness intervention design should be considered before we can conduct a full study. Even if the drop-out rate was acceptable, according to the feasibility criteria, a sample size calculation to a full study must consider the loss to follow-up. In the present pilot-study, there were 11 participants excluded from analysis due to low activity monitor wear time. In an attempt to increase the adherence to wear the activity monitor, participants should perhaps get the opportunity to choose the type of activity monitor, i.e. worn by the hip or by the wrist. However, using activity monitors measuring from different places of the body (wrist or hip) may affect the ability to conduct a correct analysis of the measurements. In a modified study, comparing the three groups (PAP, mindfulness and combination), we may need to adjust what time of day the mindfulness courses are arranged, and perhaps adjust the length of time for the daily exercise. Spending 20 min per day doing mindfulness may be hard to fit into one’s ordinary schedule and even more difficult to combine with increased physical activity.

#### Intervention outcome

The main intervention outcome was to compare differences in change over time of physical activity level between three groups: PAP, mindfulness and a combination containing both PAP and mindfulness. The results showed no differences between the groups regarding activity monitor measurements. Neither were there any differences in change of self-reported activity. We did not find any indications that mindfulness alone increased the percentage of time in physical activity, according to the data from the activity monitors. The combination seemed to increase LIPA, self-reported physical activity and SRH more than the other interventions in within-group comparisons, even if there was no difference in change between the groups.

Even if PAP and the combination seemed to increase self-reported physical activity more than mindfulness alone, the changes were small. The 2.4 percentage points increase of LIPA at 6 months compared with baseline in the combination group represented about 15 min more per day on average, based on a count with an activity monitor wear time of 600 min per day. Nevertheless, the small decrease in sedentary behaviour and increase in physical activity during the short follow-up can be seen as a positive outcome. With a longer follow-up, we may have seen a bigger change, considering that it is a major life challenge to change from being mostly sedentary to being more active. On the other hand, previous research has indicated that the self-reported effect of PAP is most pronounced during the first 3 months [36].

The discrepancy between the activity monitoring and the self-reported activity seen in the present study may be explained by low physical activity in the week when measured with an activity monitor, and thus not representative for the physical activity in an average week for the patient. However, it is a known fact that the self-reported activity level increases more over time compared to objective measurements, especially with repeated measurements [31, 40]. PAP is, at present, in the Swedish healthcare system, the only accessible tool for motivating inactive people to increase their overall activity level. PAP has indeed shown effectiveness according to Onerup et al. [11], but the findings in our study could not confirm this effect with activity monitors. Our results are consistent with a previous study [12], which failed to detect any significantly increased MVPA among patients who received PAP. Since using PAP in healthcare is time-consuming, it is important to examine if the method is effective. Therefore, larger controlled trials with PAP and activity monitors are needed in order to evaluate the effect. To obtain the participants’ true activity pattern, it is important to complete self-reported activity with activity monitors, perhaps over several weeks.

A noteworthy finding is the increase in units of SRH within all groups (Table 2; Fig. 7), which can depend on the same fact as other self-reported values that increase with repeated measurements [31, 40]. Both mindfulness [18, 41] and physical activity [42, 43] have been associated with increased levels of SRH; thus, this may be an explanation of the increased SHR in all groups.

We invited all patients, who reported themselves as physically inactive regardless of diagnosis, thus representing a usual cohort of patients in a Swedish primary health care clinic. It is possible that the results might have been different if we included a more specified group of patients. Hence, our results suggest that mindfulness may have a motivating effect. However, the small tendencies need to be confirmed by a larger study.

### Strengths

This is one of the first randomised trials with the Swedish PAP model and mindfulness aiming at a broad primary health care population with objective measurements of physical activity. According to the baseline activity monitor data, we managed to capture the most sedentary patients with a low percentage of physical activity, which was the aim. The high recruitment rate and low dropout rate indicates that patients are interested in participating in these types of studies, and thus a marker for the possibility to obtain enough participants in a bigger study with the same aim as the present study.

### Limitations

This pilot study is underpowered compared with the planned full study, which can be the reason that we failed to show significant differences between the groups regarding the activity monitor measured results. The limitation with the ACTi Graph GT1X activity monitor is that it only measures cardiorespiratory training and not other physical activities such as weightlifting, biking and swimming. Low compliance in wearing the activity monitor also compromised the reliability of the results (Table 2).

## Conclusions

The study design needs adjustment for the mindfulness intervention design before a fully scaled study can be conducted. The combination of PAP and mindfulness may increase physical activity and SRH more than PAP or mindfulness alone.

## Supplementary Information

**Additional file 1.** Table presenting differences in baseline values between dropouts and those who continued.

**Additional file 2.** Table presenting number of days with activity monitor wear time of 600 minutes or more per day.

**Additional file 3.** Intercept (adjusted baseline value) and changes from baseline to 3 and 6 months in the three groups using mixed effect models. Containing individuals with at least 1 valid day (600 minutes activity monitor wear time per day).

**Additional file 4.** Intercept (adjusted baseline value) and changes from baseline to 3 and 6 months in the three groups using mixed effect models. Containing analysis of BMI, total cholesterol, low-density cholesterol, high-density cholesterol, triglycerides, diastolic- and systolic blood pressure, insomnia severity scale and five facets of mindfulness questionnaire. Individuals with at least 4 valid days (600 minutes activity monitor wear time per day).

**Additional file 5.** Change in kilograms in the three groups over time. Differences between and within the groups are estimated by a mixed effect model.

**Additional file 6.** Change in BMI (kg/m^2^) in the three groups over time. Differences between and within the groups are estimated by a mixed effect model.

## Data Availability

The datasets generated and analysed during the current study are not publicly available due to confidentiality for patients due to small study size but are available from the corresponding author.

## Abbreviations

PAP: Physical activity on prescription
WHO: World Health Organization
ISI: Insomnia Severity Index
FFMQ: Five Facets of Mindfulness Questionnaire
LIPA: Light physical activity
MVPA: Moderate to vigorous physical activity MBSR Mindfulness-Based Stress Reduction
MBCT: Mindfulness-Based Cognitive Therapy
