# Supplementary material for: Effect of mindfulness on physical activity in primary healthcare patients: a randomised controlled trial pilot study
Source: Pilot Feasibility Stud. 2021 Mar 17;7:70. doi: 10.1186/s40814-021-00810-6 (PMC7968363; doi:10.1186/s40814-021-00810-6)
Supplement: Supplementary file 1 — Additional file 1. Table presenting differences in baseline values between dropouts and those who continued. [file 40814_2021_810_MOESM1_ESM.docx]

Additional file 1.
Differences in baseline values between dropouts and those who continued to six months follow-up

| **Variables** | Dropout before 3months | Those who continued | P |
| --- | --- | --- | --- |
| **Age**  No  Median  range | 18  51  43–62 | 70  55  41–65 | 0.18 |
| **Sex** (%)  Male No  Female No | 5 (28)  13 (72) | 19 (27)  51 (73) | 0.96 |
| **Weight** kg  No  Mean  SD | 18  95.2  17.3 | 70  86.7  18.4 | 0.06 |
| **BMI** kg/m^2^  No  Mean  SD | 18  32.5  6.1 | 70  30.2  5.2 | 0.07 |
| **Systolic blood pressure** mmHg  No  Median  range | 18  128  110–160 | 70  130  80–160 | 0.82 |
| **Diastolic blood pressure** mmHg  No  Median  range | 18  80  70–100 | 70  80  60–95 | 0.06 |
| **Cholesterol** mmol/l  No  Mean  SD | 18  5.5  1.1 | 70  5.4  .98 | 0.76 |
| **Low density cholesterol** mmol/l  No  Mean  SD | 18 3.9  0.96 | 70  3.6  .97 | 0.29 |
| **High density cholesterol** mmol/l  No  Median  range | 18  1.3  0.89–2.4 | 70  1.5  0.47–3.1 | 0.06 |
| **Triglycerides** mmol/l  No  Mean  SD | 18  1.7  .75 | 70  1.7  1.0 | 0.50 |
| **ISI***  No  Median  range | 17  10  1–26 | 70  10  0–27 | 0.73 |
| **FFMQ ****  No  Median  range | 17  105  80–128 | 70  102  82–129 | 0.41 |
| **SRH*****  No  Median  range | 17  3  2–4 | 70  3  1–5 | 0.76 |
| **Self-reported leisure time** **activity** (0-6)  No  Median  range | 17  1  1–5 | 70  2  1–5 | 0.92 |
| **Self-reported daily activity** (1-7)  No  Median  range | 17  4  2–7 | 70  3  1–6 | 0.66 |
| **Percentage time Sedentary** (activity monitor data) No  Mean SD | 13 65.8 10.1 | 65 66.2 8.0 | 0.94 |
| **Percentage time in LIPA******(activity monitor data) No  Mean SD | 13 31.1 8.6 | 65 30.9 7.3 | 0.98 |
| **Percentage time in MVPA*******(activity monitor data**)** No  Median range | 13 2.1 0.1–8.6 | 65 2.4 0.2–9.1 | 0.63 |

Data are presented as mean values and standard deviation (SD) for normally distributed variables, and as median and range for variables with skewed distribution and variables based on nominal scales. ****LIPA: light physical activity, *****MVPA: moderate to vigorous physical activity, *ISI: insomnia severity index, **FFMQ: Five facets of mindfulness questionnaire, ***SRH: self-rated health.
