# Supplementary material for: Effect of mindfulness on physical activity in primary healthcare patients: a randomised controlled trial pilot study
Source: Pilot Feasibility Stud. 2021 Mar 17;7:70. doi: 10.1186/s40814-021-00810-6 (PMC7968363; doi:10.1186/s40814-021-00810-6)
Supplement: Supplementary file 2 — Additional file 2. Table presenting number of days with activity monitor wear time of 600 minutes or more per day. [file 40814_2021_810_MOESM2_ESM.docx]

Additional file 2.
Number of days with activity monitor wear time of 600 minutes or more per day.

| **Timepoint** | **> 4 days** | **> 5 days** | **> 6 days** | **> 7 days** | **No.** | **Missing No.** |
| --- | --- | --- | --- | --- | --- | --- |
| **Baseline** | 70  86.4% | 66  81.5% | 57  70.4% | 49  60.5% | 81 | 7 |
| **3 months** | 60  84.5% | 53  74.6% | 52  73.2% | 41  57.7% | 71 | 8 |
| **6 months** | 51  82.3% | 46  74.2% | 37  59.7% | 25  40.3% | 62 | 8 |
