# Supplementary material for: Effect of mindfulness on physical activity in primary healthcare patients: a randomised controlled trial pilot study
Source: Pilot Feasibility Stud. 2021 Mar 17;7:70. doi: 10.1186/s40814-021-00810-6 (PMC7968363; doi:10.1186/s40814-021-00810-6)
Supplement: Supplementary file 3 — Additional file 3. Intercept (adjusted baseline value) and changes from baseline to 3 and 6 months in the three groups using mixed effect models. Containing individuals with at least 1 valid day (600 minutes activity monitor wear time per day). [file 40814_2021_810_MOESM3_ESM.docx]

Additional file 3.
Intercept (adjusted baseline value) and changes from baseline to 3 and 6 months in the three groups using mixed effect models. Individuals with at least 1 valid day (600 minutes activity monitor wear time per day).

| Outcome | Adjusted baseline value | 3 months change | 6 months change | Mean difference between groups over time  (95% CI) |
| --- | --- | --- | --- | --- |
| Sedentary percentage* |  |  |  | -0.75  -1.80 ; 0.31 |
| PAP | 66.5 | 1 | -0.8 |  |
| Mindfulness | 65.5 | 0.5 | 1 |  |
| Combination | 66.8 | 0.0 | -3.6 |  |
| LIPA percentage* |  |  |  | 0.59  -0.38 ; 1.57 |
| PAP | 30.7 | 0.5 | 0.7 |  |
| Mindfulness | 31.7 | -0.6 | -0.7 |  |
| Combination | 30.1 | -0.9 | 3.1 |  |
| MVPA percentage* |  |  |  | 0.14  -0.20 ; 0.47 |
| PAP | 2.8 | -0.5 | 0.0 |  |
| Mindfulness | 2.9 | 0.0 | -0.3 |  |
| Combination | 3.0 | 0.9 | 0.5 |  |
| Leisure time activity** |  |  |  | 0.16  -0.06 ; 0.39 |
| PAP | 1.63 | 1.1 | 1.3 |  |
| Mindfulness | 1.85 | 0.7 | 0.6 |  |
| Combination | 1.79 | 1.6 | 1.8 |  |
| Daily activity ** |  |  |  | -0.05  -0.27 ; 0.17 |
| PAP | 3.42 | 1.1 | 1.3 |  |
| Mindfulness | 3.54 | 0.5 | 0.8 |  |
| Combination | 3.46 | 1.0 | 1.0 |  |
| Weight(kg) |  |  |  | 0.10  -0.33 ; 0.52 |
| PAP | 90.9 | -1.7 | -2.0 |  |
| Mindfulness | 86.4 | -0.6 | -0.8 |  |
| Combination | 84.7 | -1.0 | -1.5 |  |
| BMI (kg/m^2^) |  |  |  | 0.11  -0.14 ; 0.35 |
| PAP | 31.6 | -0.6 | -1.2 |  |
| Mindfulness | 30.3 | -0.2 | -0.3 |  |
| Combination | 29.1 | -0.5 | -0.7 |  |
| Total cholesterol (mmol/l) |  |  |  | -0.02  -0.16 ; 0.12 |
| PAP | 5.20 | -0.21 | -0.07 |  |
| Mindfulness | 5.68 | -0.35 | -0.08 |  |
| Combination | 5.41 | -0.32 | -0.16 |  |
| Low density cholesterol (mmol/l) |  |  |  | 0.02  -0.11 ; 0.15 |
| PAP | 3.42 | -0.20 | -0.05 |  |
| Mindfulness | 3.84 | -0.36 | -0.03 |  |
| Combination | 3.67 | -0.15 | 0.02 |  |
| High density cholesterol (mmol/l) |  |  |  | -0.01  -0.04 ; 0.02 |
| PAP | 1.55 | -0.04 | -0.04 |  |
| Mindfulness | 1.50 | 0.02 | 0.03 |  |
| Combination | 1.51 | -0.08 | -0.09 |  |
| Triglycerides (mmol/l) |  |  |  | -0.04  -0.16 ; 0.08 |
| PAP | 1.40 | 0.05 | 0.05 |  |
| Mindfulness | 1.82 | -0.10 | 0.07 |  |
| Combination | 1.60 | -0.03 | -0.09 |  |
| Systolic Blood pressure (mmHg) |  |  |  | 2.29  0.19 ; 4.39 |
| PAP | 128.5 | -5.4 | -4.9 |  |
| Mindfulness | 122.4 | 0.89 | 2.6 |  |
| Combination | 125.7 | -2.6 | 4.6 |  |
| Diastolic blood pressure (mmHg) |  |  |  | 1.25  -0.30 ; 2.80 |
| PAP | 79.5 | -1.5 | 0.3 |  |
| Mindfulness | 79.0 | 0.2 | 1.2 |  |
| Combination | 76.6 | -1.5 | 5.4 |  |
| ISI ** |  |  |  | -0.27  -1.04 ; 0.49 |
| PAP | 8.8 | -0.9 | -1.0 |  |
| Mindfulness | 12.5 | -0.5 | -0.5 |  |
| Combination | 10.9 | -1.2 | -2.0 |  |
| FFMQ ** |  |  |  | 0.18  -0.91 ; 1.27 |
| PAP | 105.3 | 0.9 | 1.9 |  |
| Mindfulness | 105.2 | 0.6 | -0.8 |  |
| Combination | 100.9 | 3.3 | 2.3 |  |
| SRH ** |  |  |  | 0.05  -0.06 ; 0.17 |
| PAP | 3.3 | 0.1 | 0.1 |  |
| Mindfulness | 3.1 | 0.4 | 0.4 |  |
| Combination | 3.3 | 0.4 | 0.4 |  |

* Percentage of mean time measured by activity monitor
**Self-reported measurements: leisure time activity on a scale from 1= 0 minutes per week, 6 => 120 min/week. Daily activity on a scale 1= 0 minutes per week, 7=>300 min per week. insomnia severity index (isi) 0-28 points, five facets of mindfulness questionnaire (FFMQ) 39-195 points. Self-rated health (srh) 1-5 points
