# Supplementary material for: Effect of mindfulness on physical activity in primary healthcare patients: a randomised controlled trial pilot study
Source: Pilot Feasibility Stud. 2021 Mar 17;7:70. doi: 10.1186/s40814-021-00810-6 (PMC7968363; doi:10.1186/s40814-021-00810-6)
Supplement: Supplementary file 4 — Additional file 4. Intercept (adjusted baseline value) and changes from baseline to 3 and 6 months in the three groups using mixed effect models. Containing analysis of BMI, total cholesterol, low-density cholesterol, high-density cholesterol, triglycerides, diastolic- and systolic blood pressure, insomnia severity scale and five facets of mindfulness questionnaire. Individuals with at least 4 valid days (600 minutes activity monitor wear time per day). [file 40814_2021_810_MOESM4_ESM.docx]

Additional file 4.
Intercept (adjusted baseline value) and changes from baseline to 3 and 6 months in the three groups using mixed effect models. Individuals with at least 4 valid days (600 minutes activity monitor wear time per day). ISI: insomnia severity index, FFMQ: five facets of mindfulness questionnaire.

| Outcome | Adjusted baseline value | 3 months change | 6 months change | Mean difference between groups over time  (95% CI) |
| --- | --- | --- | --- | --- |
| BMI (kg/m^2^) |  |  |  | 0.21  -0.06 ; 0.49 |
| PAP | 31.7 | -0.7 | -1.5 |  |
| Combination | 29.0 | -0.2 | -0.5 |  |
| Mindfulness | 30.0 | -0.1 | -0.3 |  |
| Total cholesterol (mmol/L) |  |  |  | -0.08  -0.24 ; 0.07 |
| PAP | 5.23 | -0.14 | -0.06 |  |
| Combination | 5.54 | -0.43 | -0.31 |  |
| Mindfulness | 5.70 | -0.51 | -0.27 |  |
| Low density cholesterol (mmol/L) |  |  |  | -0.01  -0.15 ; 0.13 |
| PAP | 3.47 | -0.15 | -0.02 |  |
| Combination | 3.79 | -0.25 | -0.06 |  |
| Mindfulness | 3.87 | -0.52 | -0.24 |  |
| High density cholesterol (mmol/L) |  |  |  | -0.3  -0.06 ; 0.00 |
| PAP | 1.57 | -0.03 | 0.02 |  |
| Combination | 1.51 | -0.08 | -0.14 |  |
| Mindfulness | 1.49 | -0.002 | 0.04 |  |
| Triglycerides (mmol/L) |  |  |  | -0.06  -0.17 ; 0.06 |
| PAP | 1.41 | -0.01 | 0.07 |  |
| Combination | 1.67 | -0.08 | -0.17 |  |
| Mindfulness | 1.70 | 0.11 | -0.06 |  |
| Systolic Blood pressure (mmHg) |  |  |  | 1.88  -0.51 ; 4.27 |
| PAP | 128.0 | -5.4 | -5.2 |  |
| Combination | 125.1 | -2.7 | 3.0 |  |
| Mindfulness | 120.1 | 0.3 | 4.7 |  |
| Diastolic blood pressure (mmHg) |  |  |  | 0.72  -0.99 ; 2,43 |
| PAP | 79.5 | -1.9 | 0.2 |  |
| Combination | 76.3 | -1.7 | 3.3 |  |
| Mindfulness | 77.6 | -0.9 | 2.3 |  |
| ISI |  |  |  | -0.27  -1.12 ; 0,58 |
| PAP | 9.2 | 0.2 | -1.2 |  |
| Combination | 10.7 | -1.4 | -2.0 |  |
| Mindfulness | 11.3 | -1.3 | -0.2 |  |
| FFMQ |  |  |  | 0.50  -0.76 ; 1.75 |
| PAP | 105.1 | 1.7 | 1.8 |  |
| Combination | 100.6 | 3.5 | 3.3 |  |
| Mindfulness | 105.9 | 1.3 | -1.3 |  |
