# Supplementary material for: Effect of mindfulness on physical activity in primary healthcare patients: a randomised controlled trial pilot study
Source: Pilot Feasibility Stud. 2021 Mar 17;7:70. doi: 10.1186/s40814-021-00810-6 (PMC7968363; doi:10.1186/s40814-021-00810-6)
Supplement: Supplementary file 6 — Additional file 6. Change in BMI (kg/m2) in the three groups over time. Differences between and within the groups are estimated by a mixed effect model. [file 40814_2021_810_MOESM6_ESM.docx]

Additional file 6.
Change in BMI (kg/m^2^) in the three groups over time.
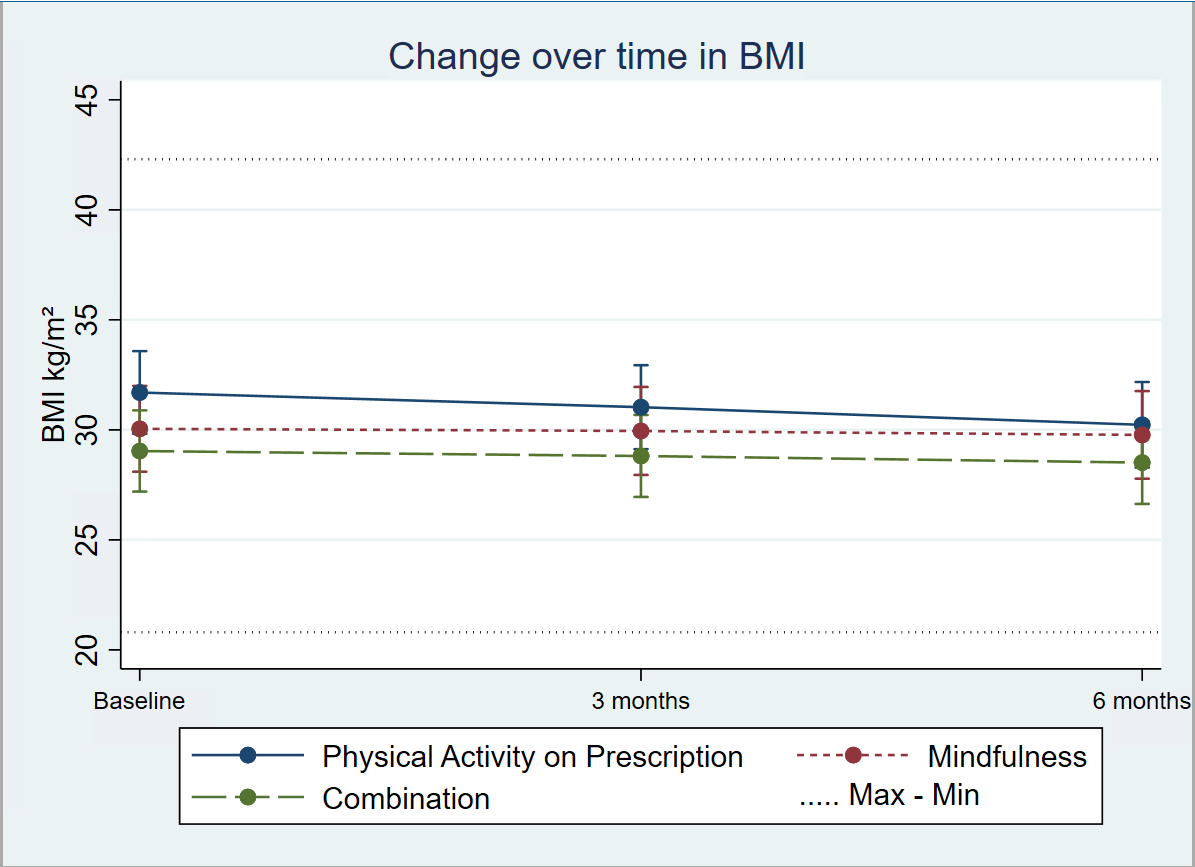

Differences between and within the groups are estimated by a mixed effect model. Max= 42.3, Min=20.8.
